# Prevalence and factors associated with percutaneous injuries and splash exposures among health-care workers in a provincial hospital, Kenya, 2010

**DOI:** 10.11604/pamj.2013.14.10.1373

**Published:** 2013-01-06

**Authors:** Everline Muhonja Mbaisi, Zipporah Ng'ang'a, Peter Wanzala, Jared Omolo

**Affiliations:** 1 Jomo Kenyatta University of Agriculture and Technology, Field Epidemiology and Laboratory Training Programme and Ministry of Public Health and Sanitation, Kenya; 2 Institute of Tropical Medicine and Infectious Diseases, Jomo Kenyatta University of Agriculture and Technology; 3 Kenya Medical Research Institute, Center for Public Health Research, Kenya

**Keywords:** Health-care, occupational exposure, blood, body fluids, blood-borne pathogens, HIV

## Abstract

**Introduction:**

Accidental occupational exposure of healthcare workers to blood and body fluids after skin injury or mucous membrane contact constitutes a risk for transmission of blood-borne pathogens. Such pathogens include Human Immunodeficiency Virus (HIV), Hepatitis B virus (HBV) and Hepatitis C virus (HCV). We conducted a study to determine the prevalence and associated factors for percutaneous injuries and splash exposures among health-care workers in Rift Valley provincial hospital.

**Methods:**

A cross-sectional study was carried out from October to November 2010. Self reported incidents, circumstances surrounding occupational exposure and post-exposure management were sought by use of interviewer administered questionnaire. Descriptive, bivariate and multiple logistic regression (forward stepwise procedure) analyses were performed. The level of significance was set at 0.05.

**Results:**

Twenty five percent of health-care workers interviewed (N = 305) reported having been exposed to blood and body fluids in the preceding 12 months. Percutaneous injuries were reported by 19% (n = 305) and splash to mucous membrane by 7.2%. Higher rates of percutaneous injuries were observed among nurses (50%), during stitching (30%), and in obstetric and gynecologic department (22%). Health workers aged below 40 years were more likely to experience percutaneous injuries (OR= 3.7; 95% CI = 1.08-9.13) while previous training in infection prevention was protective (OR= 0.52; 95% CI = 0.03-0.90). Forty eight percent (n = 83) reported the incidents with 20% (n = 83) taking PEP against HIV.

**Conclusion:**

Percutaneous injuries and splashes are common in Rift Valley Provincial hospital. Preventive measures remain inadequate. Health institutions should have policies, institute surveillance for occupational risks and enhance training of health care workers.

## Introduction

Occupational exposure to blood or other body fluids in healthcare facilities constitutes a significant risk of transmission of HIV and other blood borne pathogens to healthcare workers. HIV/AIDs in particular is a major threat in the workplace [1]. Occupational risks associated with exposure affects the quality of care delivered as well as health-care workers safety and well being [2]. As a result exposed workers experience significant fear, anxiety and emotional distress that can result in occupational and behavioral changes [3].

The World Health Organization (WHO) estimates that 3 million percutaneous exposures occur annually among 35 million HCW globally; over 90% occurring in resource constrained countries [4]. Health-care workers in Africa suffer two to four needle-stick injuries per year on average [5], with Nigeria, Tanzania and South Africa reporting 2.10 injuries per HCW on average [6]. Worldwide occupational exposure accounts for 2.5% of HIV cases and 40% of Hepatitis B and C cases among HCWs [7]. Each year as a consequence of occupational exposure, an estimated 66,000 Hepatitis B, 16,000 Hepatitis C and up-to 1,000 HIV infections occur among HCWs. These infections are preventable through infection control measures which significantly reduce the risk of HIV and Hepatitis transmission among health workers [8].

Most developing countries, Kenya included, may not have surveillance for occupational exposure to blood and body fluids, hence limiting estimation of the exact magnitude of such accidents. We determined the prevalence of percutaneous injuries and splashes, described the circumstances surrounding the exposures and determined the associated factors among healthcare workers in a provincial hospital in Nakuru, Kenya.

## Methods

### Study design

We conducted a cross sectional study on prevalence and associated factors for percutaneous injuries and splashes among a random sample of health-care workers from 8th October 2010 to December 2010.

### Study setting

The study was conducted in a large public hospital in Nakuru, Kenya. Rift valley provincial general hospital (RVPGH) is a level five public hospital in Nakuru central district of Rift valley province. RVPGH is a referral hospital offering general services, with a catchment population of about 500,000, bed capacity of 588 and 60 cots, and an average monthly bed occupancy of 110%.

### Study population

The study population consisted of health-care workers who came into contact with patients, or were potentially exposed to body fluids from patients while attending to or handling samples from patients. These healthcare workers included resident doctors and intern-doctors, clinical officers, nurses, laboratory personnel, mortuary attendants, housekeeping staff and students (nurses, clinical officers, lab technicians/technologists). Those who were present at the time of data collection were recruited.

### Sampling procedure and sample size determination

A minimum sample of 246 was determined using modified Cochran formula 1977 [9], with finite population correction, based on the assumptions of 17.2% prevalence of sharps injuries among HCWs [10], 95% confidence interval, 5% absolute precision. We used stratified sampling method to recruit staff into the study, based on the various categories of staff, who were allocated proportionally according to the size of each strata (Number selected from each stratum= Population in stratum *(n/N).

### Data collection tools and methods

Data was collected using a standard semi-structured questionnaire adopted from Centre for Disease Control and Prevention workbook for designing sharp prevention programme [11]. The questionnaires were pre-tested and appropriate modifications made. Four Interviewers who were trained prior to data collection, conducted field work from 8th October to 6th November 2010. Information was collected on basic demographics, occupational data, frequency and nature of exposures and risk factors for occupational exposures.

### Data management and analysis

Data was coded during collection. We used Epi-info version 3.5.1 (CDC, Atlanta, USA) for data entry and analysis. We carried out descriptive analysis to determine frequencies, proportions and means. We determined the prevalence of percutaneous injuries and splashes, frequency of reporting and proportion of those who received PEP. We used bivariate analysis to determine measure of association (odds ratio) between occupational exposure and associated factors. Factors that were found to be significantly associated with the outcome, at P-value equal to or less than 0.25 were entered in multivariate logistic regression model (forward stepwise procedure). Statistical significance of the associations was determined by Chi-square, with a P-value of less than 0.05 considered significant.

### Ethical issues

Approval and clearance for this study was received from KEMRI national Scientific and Ethical Review Board, and hospitals' administrative authorities, before commencement of the study. Written Informed consent was sought and obtained from the participants before administration of the questionnaires. Participation was voluntary.

## Results

### Demographic characteristics of respondents

Three hundred and five health-care workers were interviewed, majority were females. The ages of the study participants ranged from 19 to 56 years, with mean age of 32 years. Staff who participated in the study included doctors, nurses, clinical officers, laboratory personnel, dentists, supportive staff and students who were on duty during the study period (Table 1).

**Table 1:** Demographic characteristics of the respondents

Variable	Frequency (%), N = 305
**Sex**	
Male	106 (34.8)
Female	199 (65.2)
**Occupation**	
Doctors	25 (8.2)
Nurses	134 (43.9)
Clinical Officers	43 (14.1)
Laboratory Personnel	12 (3.9)
Dentists/Dental technologists	3 (1)
Students	40 (13.1)
Support staff	46 (15.1)
Morgue attendant	1 (0.3)
**Age-group**	
≤ 20 years	5 (1.6)
21-30 years	164 (53.8)
31-40 years	91 (29.5)
41-50 years	31 (10.2)
> 50	14 (4.9)

### Prevalence and nature of exposures

Eighty one incidents of percutaneous injuries and mucocutaneous exposures were reported by healthcare workers within the previous 12 months, the majority of whom were female (66%). Exposure was classified as percutaneous (19%, n = 305) and mucocutaneous (7.2%, n = 305). Seven persons had more than one sharp injury, with mean number of injuries of 1.14 (SD 0.43). Percutaneous injuries were more common (73%) than splash exposures to mucocutaneous membranes (27%). Four HCWs reported having had both percutaneous injuries and splash exposure to mucous membrane within the previous 12 months.

The prevalence of percutaneous injuries was high among female HCWs (21%), HCWs of age-group 31-40 (26.4%) and among HCWs with work experience of Table 2.

**Table 2:** Prevalence of occupational exposures by potential risk factors, Rift Valley Provincial General Hospital-2010

Variable	Prevalence,% (n/N)	
	Percutaneous	Splashes
**Sex**		
Male	17 (18/106)	8.5 (9/106)
Female	21 (41/199)	6.5 (13/199)
**Age-Group (years)**		
≤ 20	0	0
21-30	19.5 (32/164)	7.9 (13/164)
31-40	26.4 (24/91)	8.8 (8/91)
41-50	6.5 (2/31)	0
>50	7 (1/14)	7 (1/14)
**Years of experience**		
≤ 10	20.4 (45/221)	8.6 (19/221)
11-20	19.3 (11/57)	3.5 (2/57)
> 20	11 (3/27)	3.7 (1/27)
**Job category**		
Nurse	22 (29/134)	6 (8/134)
Doctor	14 (4/29)	10 (3/29)
Laboratory staff	25 (3/12)	17 (2/12)
Clinical Officer	26 (11/43)	7 (3/43)
Student	18 (7/40)	5 (2/40)
Supportive Staff	11 (5/46)	9 (4/46)

Clinical officers (26%) had the highest prevalence of percutaneous injuries followed by laboratory staff (25%) and nurses (22%). Laboratory personnel reported the highest prevalence of splash exposure to mucous membranes (Table 2).

Forty three (67.8%, N = 59) of the sharps injuries were superficial (scratch, little or no blood) while two (1.7%) involved deep penetration (intramuscular penetration). Thirty percent of the injuries were moderate (penetrated through the skin, wound bled). The finger was the most commonly injured site (81%), while the eye mucosa was the most frequently exposed to splashes (56%).

### Circumstances leading to occupational exposures

Various circumstances were associated with percutaneous injuries and splash exposures. These included: type of procedure, occupation, place of work, type of device and time of day. Hypodermic needle caused 23 (39%, N = 59). Suture needles (25%), phlebotomy needles (12%) and branulars (14%) were associated with occurrence of needle-pricks.

Most of the sharps injuries occurred in the obstetrics and gynecology departments (25%, N = 59) while surgery, medical and pediatric departments reported 17% (N = 59) each. Splash exposure occurred more commonly in surgery (31.8%, N = 22), casualty (18.2%, N = 22) and Obstetrics and gynecology department (13.6%, N = 22).

Overall nurses were commonly injured during injection (55.5%) and stitching (78.9%), laboratory personnel during blood specimen collection (25%) while supportive staff during environmental cleaning (57.1%). Stitching was the most common procedure during which injuries occurred, followed by blood specimen collection and handling of intravenous lines. Twenty percent (N = 25) of splashes occurred during delivery in labour ward. 29% (N = 59) of sharps injuries occurred during stitching, 19% occurred during blood specimen collection, 19% during handling of intravenous line and 15% during administration of injections. Injuries occurred more commonly during insertion, withdrawal or manipulation of needle (31.3%, N = 59). Handling uncooperative patient (22%, N = 59) and patient movement (20%, N = 59) also precipitated the occurrence of sharp injury. Other circumstances include recapping of the needle after use, causing 2 injuries (3.4%, N = 59). Majority of percutaneous injuries occurred during the day with 42.4% (N = 59) of cases occurring at morning hours and 35.6% occurring in the afternoon. However, 6.8% of injured HCWs could not recall time the injury occurred.

Splash exposure occurred during insertion/manipulation/withdrawal of needles (23%, N = 22), with 18% caused by splash from an injured artery. Other precipitants of splash exposure were handling uncooperative patient (14%), during disposal (9%), rapid gush of fluid during spontaneous rupture of amniotic membrane (9%) and accidental splash by a colleague (5%). Precipitants classified as others were rapid expulsion of fetus during delivery, dislodging blocked intravenous line and shaking specimen bottle.

### Use of protective equipment

At the time of the exposure, 98% of HCWs wore protective equipments. Double gloves were worn by 9% of the HCWs. No eye shield or face shield was worn during execution of procedures at the time splash exposures occurred. Masks were worn by only 4 (18%, N = 22) HCWs.

### Factors associated with percutaneous injuries and splash exposures

On bivariate analysis, factors that were found to be significantly associated with percutaneous injuries include having received training and age below 40 years (Table 3). HCW's occupation, gender, station and procedure were not significantly associated with exposure to sharps injuries.

**Table 3:** Factors associated with percutaneous injuries among health-care workers, Rift Valley Provincial General Hospital

Variable	Exposed	Not Exposed	OR	95% CI	P-value
**Age**					0.02*
≤ 40	56	204	3.84	1.15-12.86	
> 40	3	42			
**Gender**					0.45
Female	41	158	1.3	0.7-2.3	
Male	18	88			
**Work duration**					0.46
≤ 10 years	45	176	1.3	0.66-2.48	
> 10 years	14	70			
**Job Category**					
Nurse	29	105	1.3	0.73-2.3	0.37
Doctor	4	25	0.6	0.19-1.73	0.32
Lab personnel	3	9	1.4	0.37-5.38	0.42
Clinical officer	11	32	1.5	0.72-3.3	0.26
Student	7	33	0.9	0.36-2.07	0.75
**Trained**					
Yes	22	133	0.51	0.28-0.91	0.021*
No	37	113			
**Department/Station**					
Paediatric	10	26	1.72	0.78-3.81	0.17
Medical	10	28	1.6	0.72-3.49	0.25
Surgical	10	37	1.2	0.54-2.48	0.42
Laboratory	5	17	1.3	0.44-3.52	0.42
Casualty	3	21	0.6	0.16-2.0	0.28
Obstetrics/Gynecology	15	68	0.9	0.47-1.7	0.73

* Significant factors

The independent predictors of percutaneous injuries on multivariate analysis were age below than 40 years (aOR = 3.7, P-value = 0.034) and having been trained (aOR = 0.52, P-value = 0.029) (Table 4).

**Table 4:** Logistic regression of factors associated with percutaneous injuries among health-care workers, Rift Valley Provincial General Hospital

Variable	aOR	95% CI	P-value
Age ≤ 40	3.7	1.1-12.4	0.034
Trained	0.52	0.29-0.94	0.029

Working in surgical department was significantly associated with splash exposure to mucous membrane, on bivariate analysis (OR = 2.8, P-value = 0.036). Working in casualty (aOR = 4.05, P-value = 0.03) and surgical department (aOR = 3.5, P-value = 0.014) were found to be significantly associated with exposure in multivariate logistic regression model.

### Vaccination against Hepatitis B virus

Forty seven point five percent of respondents (N = 305) reported having started vaccination series against hepatitis B virus. However, 128 (42%, N = 305) were fully vaccinated, having received three doses of the vaccine. There was a significance difference in vaccine coverage between doctors and all other categories of HCWs (OR = 38, P-value

Reasons cited by HCWs for not having been vaccinated included vaccine not available (67%, N = 30), not aware of the need to be vaccinated (17%) and low risk perception (7%). Reasons classified as others were high cost and fear of side effects of the vaccine.

### Management of occupational exposure

Thirty one HCWs (52.5%, n = 59) reported percutaneous injuries. Source patient was identified by 91.5% (n = 81) of all cases. Nine (12%) were HIV positive, 64 (83%) were negative while the status of 4 (5%) was unknown. Source patients were not investigated for hepatitis B or hepatitis C infection. Ninety four percent of HCWs reported immediately within an hour.

Nine (31%, N = 28) indicated that they thought the exposure material was noninfectious, while 3 (10%, N = 28) were ignorant about the risk posed by the exposures. Seven percent were not aware that they should report. Other reasons included not knowing whom to report to, felt no need to report (ignored) and self management of exposure. Cleaning the injury site with running water was the most frequently used first aid measure in over 80% (N = 59) of HCWs injured. Other measures used for immediate management included squeezing the site (3%), cleaning with hypochlorite (5%) and cleaning with methylated spirit (15%). However, 8% of HCWs did not take any action concerning the injury. For splash exposures, 19 (86%, N = 22) HCWs cleaned the site under running water while three did not take any action.

Thirty percent of the HCWs who sustained percutaneous injuries had their baseline HIV testing done. Fifteen (25.4%, N = 59) received PEP for HIV and 11 (18.6%, N = 59) received follow up care. Only 4 (18.2%, N = 22) with splashes undertook baseline HIV testing.

Reasons for not receiving PEP varied among the HCWs. In RVPGH, 59% (n = 59) of those with percutaneous injuries just ignored the incident, 9.1% were not aware of the need to take PEP. Sixty eight percent (n = 22) of HCWS with splash exposure ignored. In War Memorial, two HCWs ignored and one was not aware of PEP. Other reason for not receiving PEP included patient's HIV sero-status being negative. Having PEP was strongly associated with patient sero-status being positive (OR = 22, PV = 0.002).

## Discussion

This study revealed that occupational injuries are still common and a concern among health-care workers. The high prevalence of percutaneous injuries (19.3%) could be attributed to the fact that being a public hospital, it has a high workload, a factor identified to be associated with occurrence of occupational injuries [12]. The prevalence of 19.3% of sharps injuries is similar to a report from United Arab Emirates [13], in which 19% of HCWs reported sharps injuries in one calendar year. It is reported that in developing countries where the prevalence of HIV-infected patients is the highest in the world, the number of needle-stick injuries is also the highest [14]. However, the prevalence of splash exposure to mucous membrane was low (7%) as compared to that reported (25%) in Ethiopia [10] and 18% in a study conducted in India [8]. Other possible reasons for high prevalence of percutaneous injuries include lack of specific programme measures to address occupational challenges such as inadequate PPEs, lack of safer sharp devices, lack of information and non-adherence to standard precautions.

The prevalence of percutaneous injuries was high among those with experience less than 10 years (20.4%). Clarke et al. in their study found that the probability of ever having a needle-stick injury was inversely related to years of experience [15]. This may be attributed to inadequate skills and knowledge regarding injection safety.

Accidental exposures were more frequently reported by females (Prevalence of 21%). Despite the absence of a statistically significant association between gender and occupational exposure to blood, similar results have been previously reported [16].

Among the procedures that placed HCWs at risk of NSIs, stitching was the highest (29%), followed by blood specimen collection (19%). Cervini & Bell reported that majority of injuries among doctors occurred while stitching (46%) [17]. Situations precipitating injuries include manipulating needles (34%), patient movement (20%), recapping (3.4%), and the unsafe collection of sharps and sharps disposal (3%). This is comparable to findings in which manipulating the needle contributed to 26% and recapping 6% of injuries [11]. In another study, recapping was identified to account for 8.3% of percutaneous injuries [8].

Although all healthcare workers in contact with patients are at risk to exposure to blood and body fluids, nurses reported most percutaneous injuries (50%) and splash exposures (40%). According to other studies, nurses experience the majority of needle-stick injuries in the world including half of the exposures that occur in USA [5, 10]. Nurses are more likely to handle sharp devices and have more contact with patients.

Hypodermic needle caused 39% of percutaneous injuries. Other hollow-bore needles that caused injuries include branulars and phlebotomy needles. Overall, hollow-bore needles caused 67% of injuries. Russi et al. reported that 62% of exposures to blood and body fluids involved hollow-bore needles [18]. Hollow-bore needles have been identified as a risk factor that enhances transmission of pathogens, due to its nature of containing residual blood and other fluids and hence the most hazardous instruments among medical sharp devices [12].

Majority of exposures occurred during the morning shift (42%). This may be attributed to busy schedule at the time and the pressure among staff to complete tasks. In addition, more invasive procedures are performed in the morning. In other studies, analysis of 411 recorded exposures demonstrated that more people were exposed between 9.00 am and 11.00 a.m. [19].

Health-care workers of the age-group 31 to 40 had the highest prevalence of percutaneous injuries (26.4%). Age below 40 years was significantly associated with sharps injuries (aOR= 3.7; P-value = 0.034). This is comparable to a study conducted in Turkey in 2008 in which young age was a risk factor for occupational injuries [20]. This is possibly due to limited professional experience and the fact that young HCWs tend to be enthusiastic and aggressive in their work.

Previous training in infection prevention was protective (aOR= 0.52; P-value = 0.029).. According to a study conducted by Nsubuga et al. in Uganda, lack of training was identified as a risk factor for needle-stick injuries [21]. Training enhances awareness and improves skills among health-care workers.

Working in casualty (aOR = 4.05, P-value = 0.03) and surgical department (aOR = 3.5, P-value = 0.014) were identified as risk factors for sustaining splash exposure to mucocutaneous membrane. This is comparable to findings by Hosoglu et al. in Turkey, in which working in a surgical site was a significant factor for occupational exposure [20]. Possible explanation is that casualty is an emergency unit where procedures are carried out as urgent, while in surgical department, the kind of procedures carried out tend to predispose HCWs to splashes.

National Institute of Occupational Safety and Health, United States identifies the following as predisposing factors to needle-stick injuries; over-use of injections and unnecessary sharps, lack of supplies (disposable syringes, safer needle devices, sharps disposal containers), lack of access to, and failure to use sharps container immediately after use, poorly trained staff, needle-recapping, no engineering control, such as safer needle devices, passing instruments from hand to hand as on operating room, and lack of hazard awareness and training [22]. This is in agreement with findings from this study in which 51% of staff are untrained, facilities lack safety devices and needle recapping is still practiced.

Hepatitis B vaccination coverage among HCWs was low at 40% (fully vaccinated). According to the WHO estimates, vaccination coverage varies from 18% in Africa to 77% in Australia and New Zealand [23]. In a study conducted in 2005, Thika district Kenya, only 12.8% of HCWs were vaccinated [24]. Doctors were more likely to be vaccinated among the HCWs (OR = 38, P-value25].

There are many potential reasons for low HBV vaccine coverage, the most common being unavailability of the vaccine at the health facility. While the vaccine is available at the market at a cost, HCW have relied on provision by their institutions. However, there is a moderately good awareness among HCWs. Other potential reasons identified in our study and supported by other studies include busy schedules, lack of knowledge about severity and vaccine efficacy, and low risk perception [26]. Seven percent of health workers did not use barrier protection during execution of procedures. Skin and mucous membrane contacts can be prevented with the use of barrier precautions such as gloves, masks, gowns, and goggles. However since the greatest risk of blood-borne pathogen transmission come from percutaneous injuries, changes in techniques or use of safety devices is required. Tokars et al. noted that half of the percutaneous injuries during suturing occurred when fingers instead of instruments were used [27]. Use of personal protective equipment is critical in prevention of exposures.

In this study, over 80% of HCWs washed the injured site under running water and 54% took no action. However, a number of staff used disinfectants (hypochlorite solution or methylated spirit) to clean the site, while others squeezed the site probably due to lack of knowledge about what immediate action to take. In a study conducted by Rahul et al. in India, 60.9% of HCWs washed the site with soap and water and 14.8% took no action [28].

Forty five percent of HCWs did not report the occurrence of exposures. Unreported needle-stick and sharp injuries are a serious problem and prevent injured HCWs from receiving PEP against HIV, which is shown to be 80% effective against HIV infection. According to researchers, 40%-70% of all needle-stick injuries are unreported [14]. According to Clarke et al. in their study, only 29% of exposed respondent reported the incident [15]. Reasons for not reporting include; source thought to be non-infectious, too little time to report, lack of reporting protocol, low risk perception while other feared stigma. Moreover, underreporting may be related to unwillingness to reveal incidence or lack of motivation due to the belief that HCWs can handle the issue themselves.

Less than half of the exposed (25%) took a course of PEP against HIV/AIDS. However, this figure is high as compared to that reported in a study conducted in India where only 7.8% of HCWs took a course of PEP [28]. As most HCWs did not report the exposures, they were not evaluated for indication of PEP, therefore it is important to note that the number required to take PEP may not be exact. Over 59% of HCWs ignored the exposure. Fear of side effects has been cited as one factor against HCWs taking ARVs. A significant proportion of HCWs demonstrated lack of knowledge concerning PEP. No laboratory testing is carried out for HBV and HCV infections. Reporting injuries and documenting all blood-borne exposures are essential for having the evidence to analyze for preventive measures.

### Study limitations

We recognized certain limitations in our study. As information was self reported, misclassification of HCWs as exposed or not exposed is possible. Information on exposure was sought for the preceding 12 months; there is a possibility of recall bias among the HCWs. Due to the study design, temporal sequence cannot be ascertained.

## Conclusion

Percutaneous injuries and splashes are common in Rift Valley provincial hospital. Factors that are significantly associated with percutaneous injuries include age below 40 years and training (protective factor). Working in casualty and surgical departments were significantly associated with splash exposure. Post-exposure management is poorly adhered to with gross underreporting of the exposures.

### Recommendations

Efficient strategies to protect HCWs from occupational exposures to blood and body fluids should be identified and implemented. Facilities should establish surveillance system for registering, reporting and management of occupational injuries and exposures. Other safety measures include enhancing workers safety by providing safety devices such as auto disable/retractable needles, adhesive tapes and blunt sutures. While ensuring availability of PPEs and maintaining adequate stock is the responsibility of facility managers, HCWs have a professional obligation to use them. All HCWs should be trained, sensitized and updated on issues related to infection prevention and occupational risk reduction. Hepatitis B vaccination is recommended for HCWs and institutions should provide mandatory immunization programmes for their HCWs.
